# Development and Characterization of Functional Polylactic Acid/Chitosan Porous Scaffolds for Bone Tissue Engineering

**DOI:** 10.3390/polym14235079

**Published:** 2022-11-23

**Authors:** Miada Abubaker Osman, Nick Virgilio, Mahmoud Rouabhia, Frej Mighri

**Affiliations:** 1 Research Center for High Performance Polymer and Composite Systems (CREPEC), Montreal, QC H3T 1J4, Canada; 2 Department of Chemical Engineering, Laval University, Quebec, QC G1V 0A6, Canada; 3 Faculty of Dentistry, Laval University, Quebec, QC G1V 0A6, Canada; 4 Department of Chemical Engineering, Ecole Polytechnique of Montreal, Montreal, QC H3T 1J4, Canada

**Keywords:** open-cell scaffolds, chitosan-grafted-PLA copolymer, hydrolytic stability, bone regeneration, osteoblasts

## Abstract

In this study, we developed and characterized various open-cell composite scaffolds for bone regeneration. These scaffolds were made from Polylactic acid (PLA) as the scaffold matrix biopolymeric phase, and chitosan (CS) and chitosan-grafted-PLA (CS-g-PLA) copolymer as the dispersed biopolymeric phase. As a first step, successful grafting of PLA onto CS backbone was executed and confirmed by both FTIR and XPS. Mechanical characterization confirmed that adding CS or CS-g-PLA to the intrinsically rigid PLA made their corresponding PLA/CS and PLA/CS-g-PLA composite scaffolds more flexible under compression. This flexibility was higher for the latter due to the improved compatibility between PLA and CS-g-PLA copolymer. The hydrolytic stability of both PLA/CS and PLA/CS-g-PLA composite scaffolds inside phosphate-buffered saline (PBS) solution, as well as MG-63 osteoblast cell adhesion and proliferation inside both scaffolds, were characterized. The corresponding results revealed that PLA/CS composite scaffolds showed hydrolytic degradation due to the cationic properties of CS. However, modified PLA/CS-g-PLA scaffolds were hydrolytically stable due to the improved interfacial adhesion between the PLA matrix and CS-g-PLA copolymer. Finally, biological characterization was done for both PLA/CS and PLA/CS-g-PLA composite scaffolds. Contrarily to what was observed for uncompatibilized PLA/CS scaffolds, compatibilized PLA/CS-g-PLA scaffolds showed a high MG-63 osteoblast cell proliferation after three and five days of cell culture. Moreover, it was observed that cell proliferation increased with CS-g-PLA content. This suggests that the PLA/CS-g-PLA composite scaffolds could be a potential solution for bone regeneration.

## 1. Introduction

During the last decade, scaffold-based tissue engineering has emerged as a potential alternative for tissue grafts and organ transplantation, particularly bone and cartilage tissues [1]. The engineered scaffolds particularly serve as carriers for growing bone tissue, muscles, nerves, cartilage, ligaments, skin, and blood vessels [2]. They are typically made from biodegradable materials that gradually degrade, leaving their place to the new body tissue or organ. Depending on the type of damaged tissue or organ to be replaced, scaffolds are seeded with suitable cells in vitro, then implanted in vivo at the damage site, where cell proliferation occurs through the porous structure of the scaffold, leading to the formation of the new tissue. Therefore, to allow the cells to produce their extracellular matrix, the engineered porous scaffold should be biodegradable and biocompatible to allow cells to produce their own extracellular matrices [3].

Different classes of materials that include a variety of ceramics and synthetic or natural polymers have been successfully used to develop bone scaffolds. The most used synthetic polymeric materials are linear aliphatic polyesters that include polylactic acid (PLA), poly(glycolic acid) (PGA), and their copolymers of poly(lactic-co-glycolic acid) (PLGA) [4,5]. The degradation of these materials generally involves random hydrolysis of their ester bonds. For example, PLA degrades to form lactic acid, which is non-toxic, since it is naturally present in the body and mainly produced in small quantities (2–4 mmol/L) in muscle and red blood cells [6,7]. Porous scaffolds can also be developed by combining synthetic and natural polymers [8]. Natural polymers that are already investigated as scaffold materials include polysaccharides, such as chitosan (CS), and proteins, such as collagen [9]. Chitosan, among the most promising natural biopolymers used for bone tissue engineering, is obtained from the shells of crustaceans, a renewable source [10,11,12,13], and has a hydrophilic surface that promotes osteoblast cell adhesion and proliferation. In addition, its degradation products are non-toxic for the body [14,15]. Compared to other biopolymers, the cationic nature of CS is of great interest since it creates electrostatic interactions with negatively charged molecules, such as glycosaminoglycans, that retain growth factors secreted by colonizing bone cells [16]. Increasing the degree of deacetylation of CS largely improves cell adhesion and proliferation [17,18]. It was also reported in the literature that sulfated CS exhibits anticoagulant and antiviral properties. The regulation mechanism of sulfated chitosan affects the activity of proteins and cells strongly interacting with specific cells and biologically active substances in vivo [19,20]. 

For bone regeneration, the scaffolds’ morphology must be well-designed. An open-cell structure with a high and regular porosity is required to ensure rapid and uniform osteoblast cell proliferation. Previous studies reported that for bone tissue engineering, adequate scaffolds’ porosity should be around 90%, and that pores should provide a good interconnectivity [21]. In addition, an adequate pore size distribution should also be situated around 300 µm to ensure a homogeneous growth rate in the scaffolds’ entire volume. This porosity level was found to be optimal for osteoblast proliferation [22]. However, a high porosity reduces the mechanical properties, such as compressive strength. Growing cells may interact between each other and exert high compressive forces that could deform the scaffold, leading to an undesirable change in the shape of the final tissue structure [23]. As a result, scaffolds’ mechanical properties must be balanced against their porosity, as the two properties are intimately related. As reported in the literature, the target compressive strength is in the range of 1 to 12 MPa for cancellous bone [24,25,26], and the target compressive modulus for human bone is comprised between 4.0 and 80.0 MPA [27]. 

There are several methods to fabricate porous biodegradable synthetic polymer scaffolds, such as particulate-leaching [28,29], emulsion freeze-drying and phase separation [30], gas foaming [31,32], and even 3-D printing [33]. However, to promote cell adhesion and proliferation, most of the previous work presented in the literature needs a further solvent treatment to add biocompatible materials inside the scaffolds’ pores, such as CS, leading to unwanted residual solvents deeply trapped inside the scaffolds’ matrices [34,35]. In this study, open cell PLA/CS composite scaffolds with different CS contents were developed and characterized for their mechanical properties, thermal stability, and biocompatibility for osteoblast cell adhesion and proliferation. We particularly focused on the compatibilization of CS with the PLA matrix by grafting PLA on the CS backbone. PLA/CS-g-PLA composite scaffolds were then compared to the uncompatibilized PLA/CS scaffolds, particularly in terms of their hydrolytic degradation and ability to promote the adhesion of osteoblast cells on the surface of the pores in order to improve cell proliferation.

## 2. Materials and Methods

### 2.1. Materials Used

Polylactide (PLA) (Ingeo biopolymer 2003D (4.3 mol% D-lactide, tensile strength: 53 MPa, specific gravity: 1.24, melting temperature: 151 °C) was purchased from NatureWorks, Plymouth, MN, USA. Azodicarbonamide, ADA (CELOGEN 754A, density: 1.68 g/cm^3^ @ 25 °C, and a decomposition temperature between 164 and 180 °C) was purchased from CelChem, Naples, FL, USA. Chitosan from shrimp shells (≥75% (deacetylated)) was purchased from Sigma-Aldrich, Saint-Louis, MO, USA (product no.:C3646). N-Hydroxysuccinimide (NHS), N-(3-Dimethylaminopropyl)-N′-ethyl carbodiimide hydrochloride (M_W_: 192 g/mol, melting point: 110–115 °C), hydrochloric acid (HCl), and N, N dimethylformamide, DMF (M_W_: 73.09 g/mol; vapor pressure: 2.7 mmHg @20 °C) were purchased from Thermo Fisher Scientific, Waltham, MA, USA (products no.: 130,672, 03450, A144-212, and D119-1, respectively). Chloroform (M_W_ = 119.37) was purchased from VWR chemicals, Radnor, PA, USA (product no.: BDH1109). Sodium hydroxide was purchased from Fisher Bioreagents, Waltham, MA, USA, and Ethyl Alcohol 95% volume was purchased from Greenfield Global Inc., Brookfield, CT, USA.

### 2.2. Composite Samples Preparation and Their Foaming in Open-Cell Porous Scaffolds

#### 2.2.1. Chitosan Grafting with Polylactic Acid

The CS-g-PLA copolymer was synthesized as follows [30]: 0.8 g of CS was added to 80 mL of DMF and then stirred for 24 h. After that, 1% (*w*/*v*) of HCL solution was prepared and added to the CS/DMF solution to complete CS dissolution in DMF. Afterward, 0.8 g of PLA, 1.2 g of EDC, and 2.4 g of NHS were dissolved in 100 mL of chloroform, then the solution was added to CS/DMF solution and stirred for 48 h at ambient temperature. After that, the reaction was stopped by adjusting the solution to neutrality by adding 0.5 mol/L of sodium hydroxide solution. Finally, the final product, CS-g-PLA copolymer, was precipitated by adding excess ethanol, filtered, then dried under vacuum. By using the above protocol, the degree of substitution of PLA on CS, which is the mass concentration (%) of PLA on CS, is about 1.90 [36].

#### 2.2.2. PLA/CS and PLA/CS-g-PLA Composites Composition and Their Corresponding Preparation Steps

The following steps were used to develop the PLA/CS and PLA/CS-g-PLA composites. First, PLA pellets were grinded into powder form by cryogenic grinding and dried under vacuum at 50 °C for 12 h using a Shel Lab oven, model 1445. Then, the ADA foaming agent, dried PLA, and CS or CS-g-PLA copolymer, all in powder form, were adequately mixed for 3 min (dry mixing) using a Hamilton Beach Single-Serve Blender (model 51101BZ). Three CS or CS-g-PLA weight concentrations (5, 10, and 15 wt.%) were used, as shown in Table 1.

**Scheme 1:**
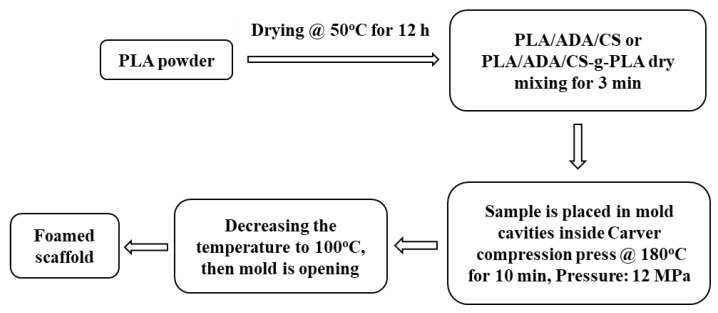
Sketch of the steps for preparing PLA/CS and PLA/CS-g-PLA open-cell scaffolds.

The corresponding open-cell PLA/CS and PLA/CS-g-PLA composite scaffolds were then obtained by compression molding for 10 min under a pressure of 12 MPa using a 15 Tons Carver automatic press (Model: Auto series 3893). The compression molding temperature was 180 °C, and the mold cavity was 6 cm × 6 cm, and 3 mm in thickness. Based on our recent work on the control of PLA foam morphology [37], to obtain an open-cell morphology with an average pore size diameter of around 300 µm needed for osteoblasts cells proliferation, a compression pressure of 12 MPa, a foaming time of 10 min, a mold opening temperature of 100 °C, and an ADA foaming agent concentration of 6.9 wt.% were used in this study and maintained constant for all the foamed samples developed. Finally, the mold was opened, and the foamed sample was cooled down to room temperature. Scheme 1 shows a sketch of the various steps of the foaming process. 

### 2.3. Characterization

#### 2.3.1. Fourier-Transform Infrared (FTIR) Spectroscopy

FTIR spectroscopy was performed, using a Nicolet Magna 860 Fourier transform spectrometer (Thermo-Nicolet, USA), to analyze and compare the chemical structures of PLA, CS, CS-g-PLA, and their corresponding PLA/CS and PLA/CS-g-PLA composites. The Happ-Genzel apodization function was used to acquire a total of 128 interferograms, coadded and Fourier-transformed to give a spectral resolution of 4 cm^−1^ in the spectral range of 4000 to 750 cm^−1^. The OMNIC (Thermo-Nicolet Co., Waltham, MA, USA) software was used for data acquisition and spectra processing (n = 4). 

#### 2.3.2. Thermogravimetric Analysis (TGA)

Thermogravimetric analysis (TGA, Q5000, TA Instruments, New Castle, DE, USA) was performed at temperatures ranging from 50 to 800 °C in a nitrogen atmosphere at a heating rate of 10 °C/min.

#### 2.3.3. Scanning Electron Microscopy (SEM) Characterization

The morphological characterization was done using a Hitachi TM3030Plus scanning electron microscope (SEM) in transversal (T) axes. The surface of the porous scaffold was exposed to liquid nitrogen to perform cryogenic fracture. The fractured surface was then coated with a thin layer of chromium before SEM characterization. 

#### 2.3.4. Mechanical Characterization in Compression of the Foamed Scaffolds

An Instron universal machine (type 5565) with the Blue Hill software (Version 2) was used to perform compressive mechanical testing of the developed scaffold samples. The tests were conducted at room temperature at a compression speed of 0.5 mm/min. Square samples (4 × 4 cm) 5 mm in thickness were used and the reported values correspond to the average of three samples. The compression modulus was calculated from the initial linear region of stress-strain curves. 

#### 2.3.5. X-ray Photoelectron Spectroscopy, XPS, Characterization

XPS characterization was conducted using a Kratos AXIS-ULTRA XPS (Kratos Analytical, Manchester, UK). The X-ray source was a monochromatic Al source operated at 300 W. The analyzer was set in the constant pass energy mode, the lens system in the “hybrid” configuration, and the aperture in the “slot” position. This ensures the highest sensitivity with an analyzed spot of approximately 700 microns × 300 microns, which is the size of the monochromatic X-ray beam. Survey scans, used for elemental analysis and apparent concentrations calculations, were recorded with a pass energy of 160 eV and a step size of 1 eV. High energy resolution spectra, used for chemical analysis, were recorded at 20 eV or 40 eV pass energy and step size of 0.05 eV or 0.1 eV, depending on the amount of each element. 

#### 2.3.6. Biological Characterization

The first step in biological characterization was the sterilization of the scaffold samples. The latter were cut manually into small pieces (0.5 × 0.5 cm and 5 mm in thickness) under a cell culture hood, then placed in 50 mL sterile tubes and immersed twice in renewed 70% ethanol solution for 60 min. At the end of this sterilization step, the scaffolds were washed twice for 15 min with sterile PBS. The washing solution was then discarded, and the scaffolds were dried under sterile conditions and stored until use.

##### Cells Adhesion Characterization (Hoechst Staining Assay)

MG-63 osteoblast cells were cultured in Dulbecco’s Modified Eagle’s medium (DMEM) supplemented with transferrin at 14.3%, 10 μg/mL of human epidermal growth factor (EGF) (Chiron Corp., Emeryville, CA, USA), 0.2 mg/mL of hydrocortisone (Calbiochem, La Jolla, CA, USA), 5 mg/mL of bovine insulin, 2 × 10^−9^ M of 3,3′,5′-triiodo-L-thyronine, and 10% fetal calf serum (NCS, fetal clone II; Hyclone, Logan, UT, USA). When the culture reached 80% confluence, cells were detached from the culture flasks by trypsin treatment, washed twice with medium, and the concentration was adjusted to 10^6^ cells/mL. Before cell seeding, PLA/CS or PLA/CS-g-PLA scaffolds containing various amounts of CS or CS-g-PLA were inserted into a low-adherence 12-well plate at one scaffold per well, then pre-incubated for 30 min in DMEM at 37 °C under a 5% CO_2_ humid atmosphere. The medium was then removed, and each scaffold was seeded with osteoblasts at a density of 2 × 10^5^ cells/scaffold in 100 μL of DMEM. Cells were allowed to adhere for 2 h, then fed with 2 mL of DMEM. The scaffolds were then incubated at 37 °C in a CO_2_-humid atmosphere for 24 h before analysis. Cell adhesion was quantitatively evaluated using methyl thiazolyl tetrazolium salt (MTS) colorimetric assay (ab197010, Abcam, Cambridge, UK). Each experiment was repeated minimally three times.

##### Cells Proliferation Quantification (MTT Assay)

MG-63 osteoblast cells were seeded at 10^5^ cells per scaffold and cultured in a CO_2_ humid atmosphere at 37 °C for either 3 or 5 days. The culture medium was refreshed each 24 h. Following each culture period of 3 and 5 days, the proliferation of osteoblast cells was assessed using the 3-(4,5-dimethylthiazole-2-yl)-2,5-diphenyltetrazolium bromide (MTT, Sigma, St. Louis, MO, USA) staining assay. This assay measures cell growth as a function of mitochondrial activity. The culture medium of each scaffold was supplemented with 1% (*v*/*v*) of a stock solution (5 mg/mL) of MTT and incubated for 3 h at 37 °C in dark conditions. The scaffolds were then washed two times with warm PBS. Following the final wash, 1 mL of a solution containing hydrochloric acid at (0.04 N) and isopropanol was added to each culture well and incubated for 15 min under shaking in dark conditions. At this step, 200 μL (in quadruplicate) of the reaction mixture was transferred to a 96-well flat-bottom plate. The absorbance was measured using a microplate reader (Model 680, Bio-Rad Laboratories Inc., Hercules, CA, USA). Results were reported as the means ± standard deviation (SD) (n = 10 scaffolds per condition).

## 3. Results and Discission

### 3.1. PLA Grafting on Chitosan

#### 3.1.1. FTIR Characterization

**Figure 1:**
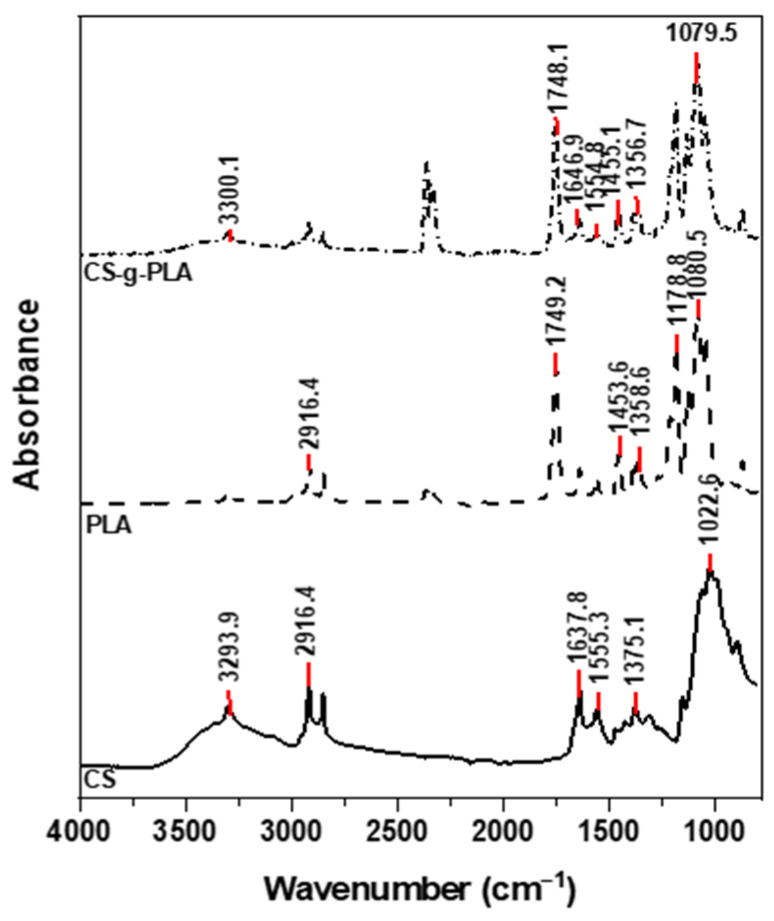
FTIR spectra of PLA, CS, and their corresponding CS-g-PLA copolymers.

FTIR was used to characterize the successful grafting of PLA onto CS backbone. Figure 1 shows the FTIR spectra of PLA, CS, and their corresponding CS-g-PLA copolymer. The CS spectrum shows two main peaks at 2916.36 and 1375.01 cm^−1^ assigned to (C-H), two peaks at 1637.77 and 1022.58 cm^−1^ respectively assigned to (C=O) and (C-O) [36], and one peak at 1555.33 cm^−1^ assigned to the amino stretch vibration of CS [38]. On the other hand, the PLA spectrum shows three main peaks at 2916.36, 1453.6, and 1358.62 cm^−1^ assigned to (C-H), one main peak at 1749.15 cm^−1^ assigned to (C=O), and two other main peaks at 1178.79 and 1080.47 cm^−1^ assigned to (C-O) [36]. Finally, the spectrum of CS-*g*-PLA clearly shows peaks attributed to PLA: the carbonyl (C=O) band of the ester group at 1748 cm^−1^, the characteristic C–O–C band at 1080 cm^−1^, the C–H band of the methyl groups at 1455 cm^−1^, and the C-H bending vibration of the methine groups at about 1360 cm^−1^. Moreover, the CS-g-PLA spectrum shows two characteristic peaks at about 1647 and 1589 cm^−1^, assigned to the amide I and amide II bonds of CS, respectively. 

#### 3.1.2. XPS Characterization of CS-g-PLA Copolymer

**Figure 2:**
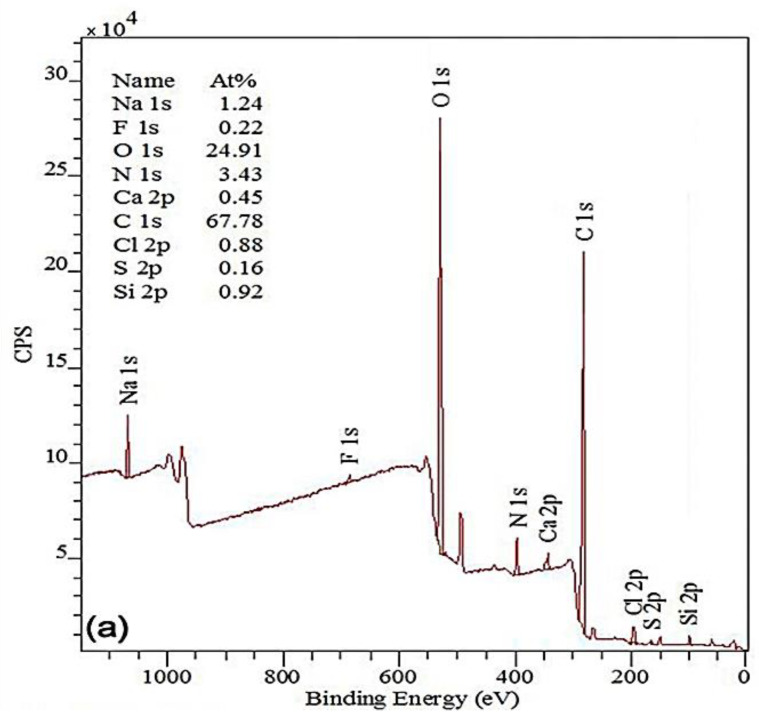

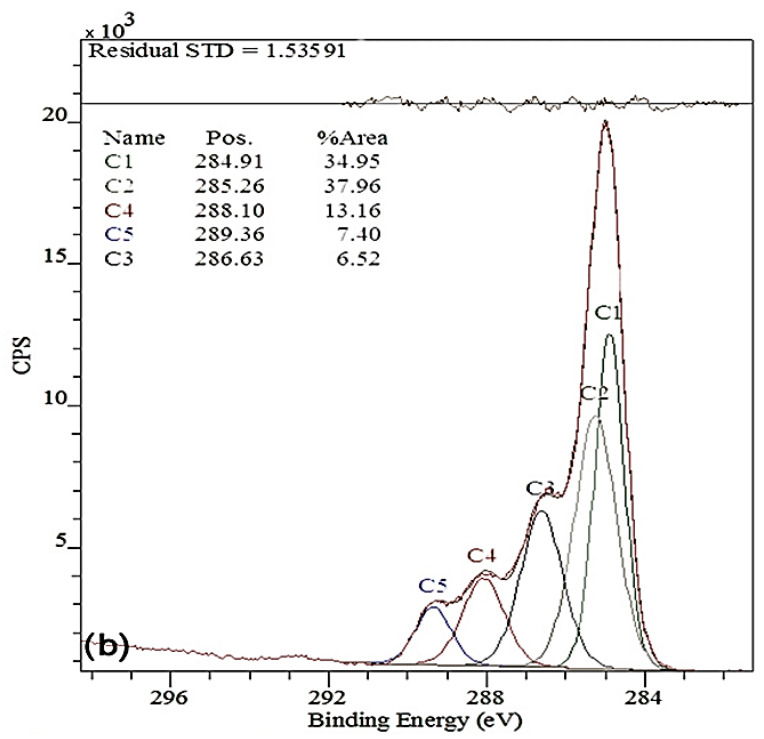
(**a**) XPS wide-scan spectrum; (**b**) C1(S) spectra of CS-g-PLA copolymers.

Figure 2 presents the XPS spectra of CS-g-PLA copolymer and provides information about copolymer elemental compositions and the type of bonding between the atoms. Figure 2a shows that carbon (C) has the highest atomic concentration (67.78 At%), i.e., CS-g-PLA skeleton is mainly made up of carbon atoms. The O atom composition is 24.91 At% and comes from the carboxyl group of PLA. The N atom represents 3.43 At% and comes from the amide bonds in CS. All the other atoms, such as Na, F, Ca, Cl, S, and Si, are present in a very small percentage and are expected to be contaminants. These results agree with those obtained by Li et al. [39] who realized the grafting of PLA on CS backbone under identical experimental protocol. Figure 2b shows the C1s spectrum of CS-g-PLA copolymer that gives information about the type of atoms bonding in the copolymer chain. The spectrum contains five peak components named C_1_ to C_5_: (i) C_1_ corresponds to C-C and C-H bonds, which are in CS, PLA, and CS-g-PLA structures; (ii) C_2_ corresponds to the C-N bond, which is in the CS structure; (iii) C_3_ corresponds to C=O, which is in CS, PLA, and CS-g-PLA structures; (iv) C_4_ corresponds to O-C-O, which is present in CS structure; and (v) C_5_ corresponds to O=C-O, which is present in PLA structure. The elemental composition and the type of atom-atom bonding discussed above confirm the successful grafting of PLA on CS.

### 3.2. Morphology of the Open Cell PLA/CS and PLA/CS-g-PLA Composite Scaffolds

**Figure 3:**
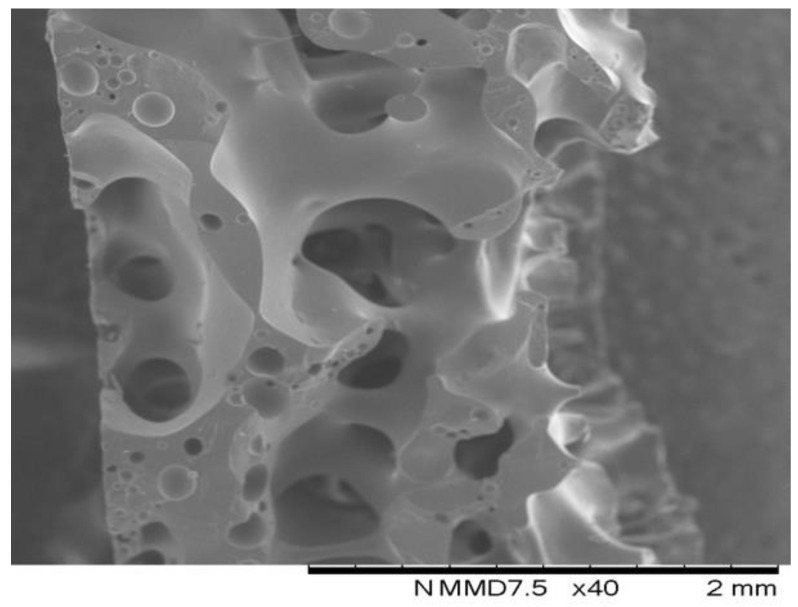
SEM of an open-cell PLA porous scaffold.

Figure 3 shows a SEM micrograph of a cryogenic fractured surface of a PLA scaffold sample. As shown, the pores inside the PLA matrix are not closed, i.e., they communicate between each other, and show an open-cell structure, which is crucial for osteoblasts proliferation. More details on parameters’ optimization to reach this morphology are presented in our previous work [37]. The average pore size (the average size of at least three measurements per cell) was around 300 µm, which is situated in the range of optimal pore size for bone regeneration [39].

### 3.3. Hydrolytic Stability of PLA/CS and PLA/CS-g-PLA Porous Composite Scaffolds

**Figure 4:**
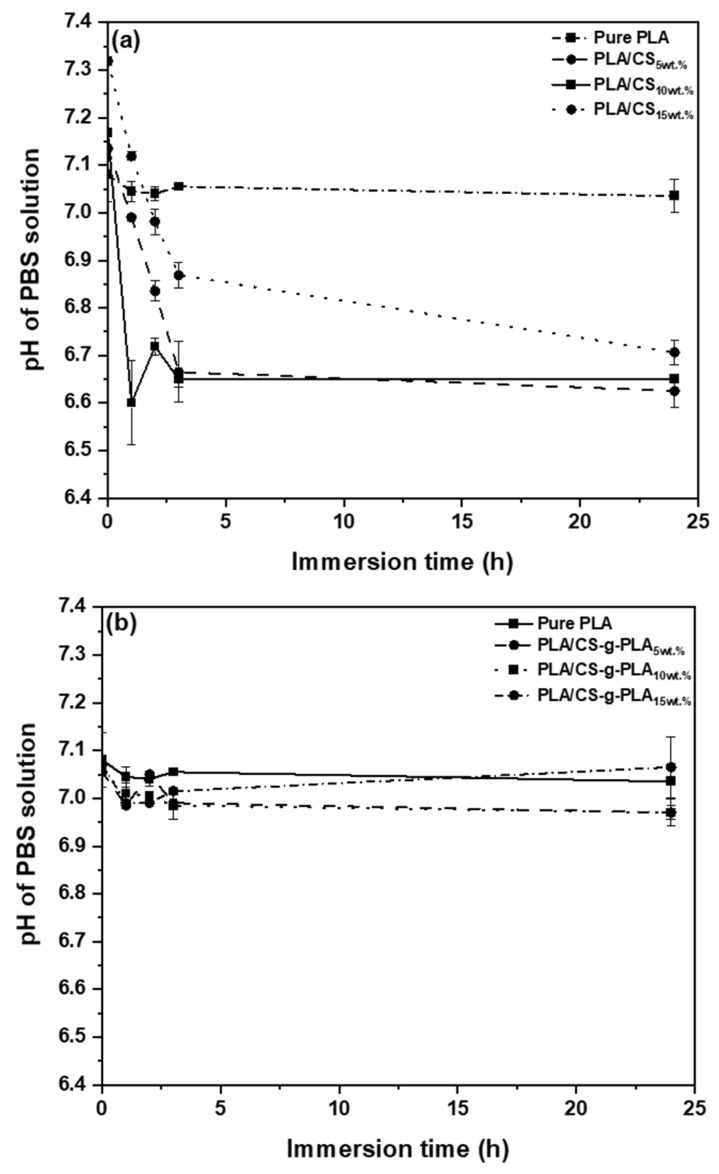
pH of PBS solution as a function of the immersion time of PLA/CS and PLA/CS-g-PLA porous scaffolds: (**a**) PLA/CS_0–15wt.%_; (**b**) PLA/CS-g-PLA_0–15wt.%_.

The pH level is an important factor to control for bone remodeling [40]. As already mentioned in the literature [40,41], the resorption of osteoclast-mediation of bone matrix is directly correlated with pH, which should be around 7.0. It was shown that acidosis in the body has a deleterious impact on bone mineralization, and osteoblastic activity increases for a pH level maintained between 7.0 and 7.6 [42,43]. Figure 4a,b show the influence of pure PLA and its corresponding PLA/CS and PLA/CS-g-PLA composite scaffolds at various CS and CS-g-PLA compositions (5, 10, and 15 wt.%), on the pH of PBS solution, as a function of immersion time. As shown in Figure 4a, for the three CS compositions studied, the immersion of PLA/CS composites in PBS solution causes a relatively significant decrease in its pH from the first day, mainly during the first four hours. This is due to the cationic properties of dissolved CS in the PBS solution, which has a low charge density and around 60% of deprotonated NH_3_^+^ groups in the neutral medium [44,45,46]. However, Figure 4b shows that pure PLA and PLA/CS-g-PLA composites are hydrolytically stable because the change in the pH of PBS solution is very small and remains between 6.95 and 7.08. This hydrolytic stability makes them appropriate for biological applications, such as bone regeneration [39,47].

### 3.4. Structures and Thermal Stability Characterizations of PLA/CS and PLA/CS-g-PLA Porous Scaffolds

**Figure 5:**
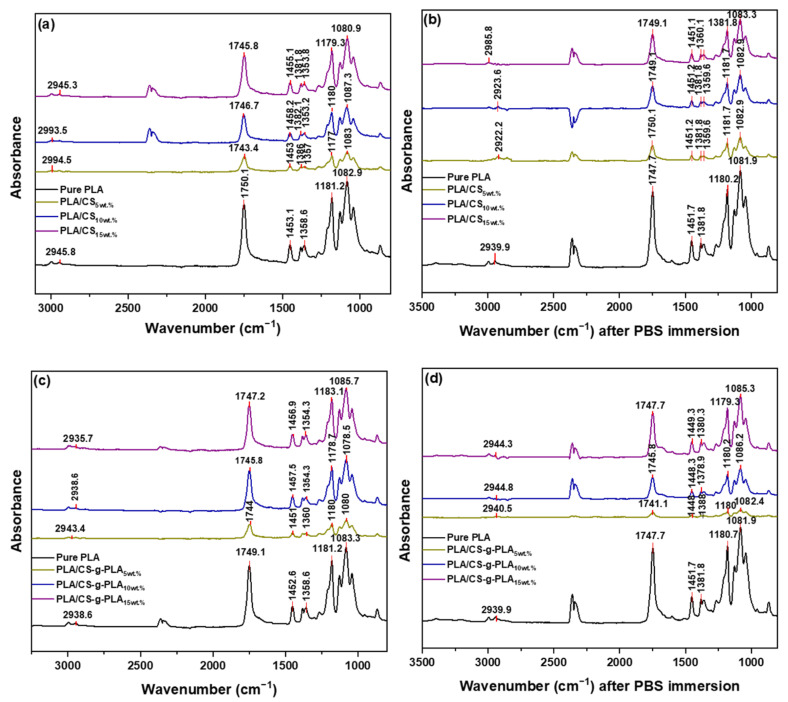
FTIR spectra of PLA/CS (**a**,**b**) and PLA/CS-g-PLA (**c**,**d**) composites, respectively, before and after their immersion in PBS solution.

Figure 5a–d shows the FTIR spectra, before and after their immersion in PBS solution, of PLA/CS and PLA/CS-g-PLA porous scaffolds with different CS and CS-g-PLA contents (5, 10, and 15 wt.%), together with the pure PLA reference sample. The figures show that for all the scaffolds studied, the spectral peaks’ intensity increases with both CS and CS-g-PLA contents. The two main peaks inside the intervals (2922.2–2994.5 cm^−1^) and (1353.2–1388cm^−1^) in the spectra of PLA/CS scaffolds (Figure 5a,b) are assigned to C-H in chitosan [36]. The three main peaks appearing inside the intervals (2922.2–2994.5 cm^−1^, 1448–1458.2 cm^−1^, and 1353.2–1388 cm^−1^) are assigned to C-H in PLA. The other PLA peaks are also appearing inside the intervals (1741.1–1750.1 cm^−1^ for C=O, 1177–1183.1 cm^−1^ and 1078.5–1087.3 cm^−1^ for C-O) [36]. On the other hand, the spectra of PLA/CS-*g*-PLA porous scaffolds (Figure 5c,d) clearly show PLA characteristic peaks inside the intervals 1743.4–1750.1 cm^−1^ assigned to the carbonyl C=O band of the ester group, inside 1080.9–1087.3 cm^−1^ assigned to C–O–C band, and those of the C–H bending vibrations of the methyl and methine groups respectively inside the intervals 1451.1–1458.2 cm^−1^ and 1353.2–1386 cm^−1^.

In conclusion, as shown in Figure 5b,d, CS and CS-g-PLA components were not extracted from the scaffolds during their immersion in the sterile PBS solution, which confirms the hydrolytic stability of the developed scaffolds, as already mentioned above in Section 3.3.

**Figure 6:**
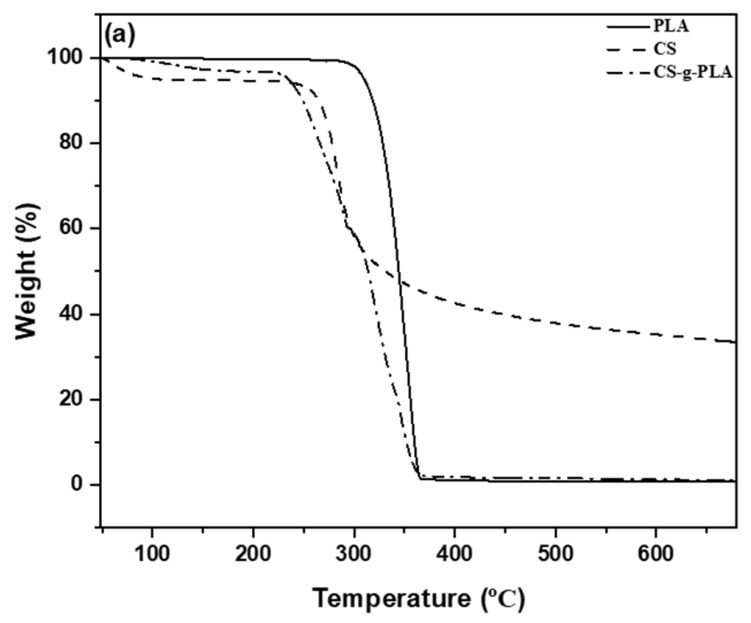

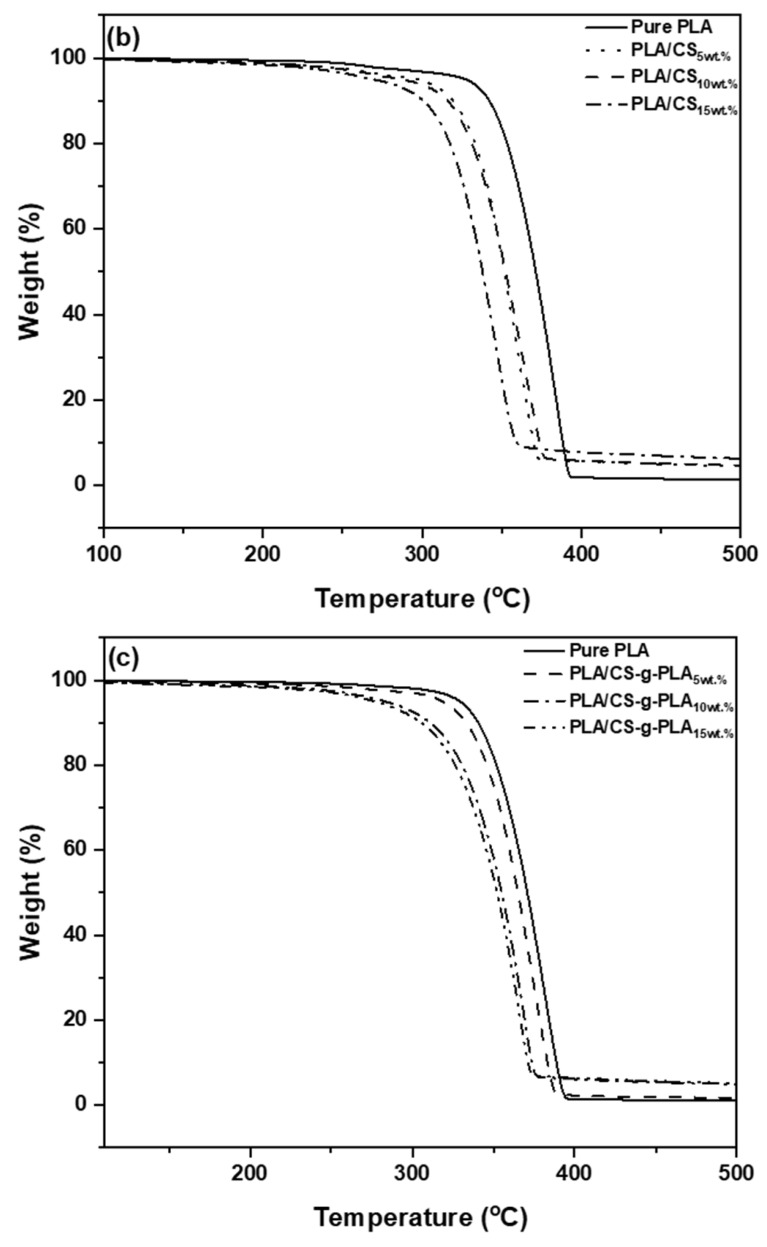
TGA curves of (**a**) PLA, CS, and CS-g-PLA; (**b**,**c**) PLA/CS and PLA/CS-g-PLA composite scaffolds at various CS and CS-g-PLA contents, respectively.

Figure 6 shows the TGA curves of PLA, CS, CS-g-PLA, and their corresponding PLA/CS and PLA/CS-g-PLA composites. Figure 6a shows that the degradation temperature of CS-g-PLA (235 °C) is lower than those of pure CS (262 °C) and pure PLA (315 °C). This is because the hydrogen bonding of CS is broken by grafting PLA chains to it. As a result, the CS’s crystallinity was reduced and disturbed, leading to the lower thermal stability of CS-g-PLA copolymer [39]. Figure 6b,c shows the same behavior for PLA/CS and PLA/CS-g-PLA composites compared to the control sample (foamed PLA). For the same reason, the degradation temperature decreases with increasing CS and CS-g-PLA up to 15 wt.%.

### 3.5. Mechanical Properties Characterization

**Figure 7:**
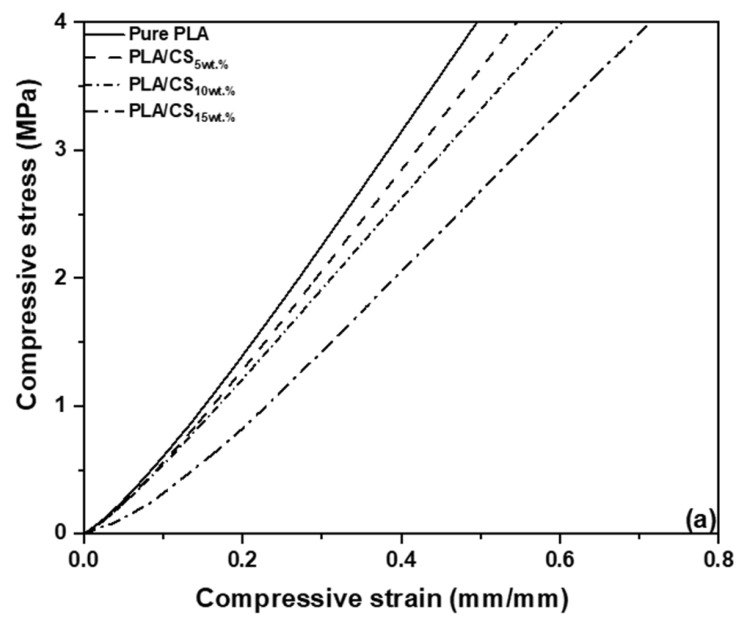

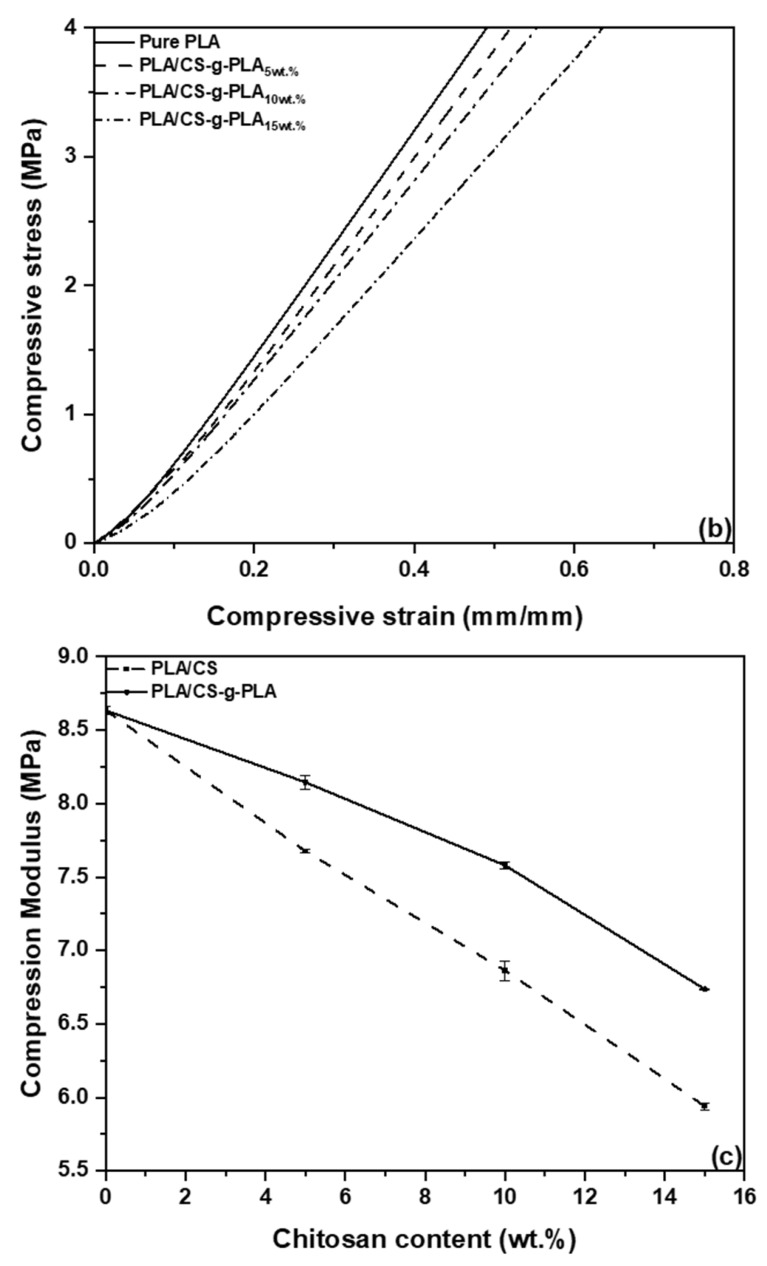
Compression stress-train curves for (**a**) PLA/CS; (**b**) PLA/CS-g-PLA porous scaffolds; and (**c**) their corresponding compression moduli, as a function of CS and CS-g-PLA contents.

Mechanical strength considerations may vary depending on whether the scaffold will be employed in vitro or in vivo. If the scaffold is going to be transplanted in vivo soon after manufacturing, its mechanical properties should ideally be similar to those of native cancellous bone to support the loads that will be applied. However, the scaffold may not require the same level of mechanical strength as natural cancellous bone if it is designed to stimulate synthetic tissue growth in vitro before implantation in vivo. This is because the scaffold will primarily serve as a supporting environment for the tissue construct’s creation [48]. Compressive stress-strain curves for both PLA/CS and PLA/CS-g-PLA foamed composite scaffolds are shown in Figure 7a,b, respectively, together with their corresponding compression moduli (Figure 7c). Figure 7a,b demonstrates that for a given compressive stress value, the composite scaffolds are more ductile than the pure PLA scaffold, which displays a brittle behavior. As a result, the addition of CS or CS-g-PLA made the scaffolds more flexible under compression. However, for all scaffolds, no plastic deformation was observed, and they all broke within the elastic stress-strain region. As shown in Figure 7c, the compression modulus for both PLA/CS and PLA/CS-g-PLA composite scaffolds decreases with increasing CS and CS-g-PLA contents, and the pure PLA scaffolds present the highest modulus. In addition, at similar chitosan contents, the modulus for the PLA/CS scaffolds is higher compared to PLA/CS-g-PLA, which is due to the lack of compatibility (weak interfacial adhesion) of PLA matrix with CS compared to its improved compatibility with CS-g-PLA that leads to better CS-g-PLA dispersion. Compared to the literature, the compression moduli for all PLA/CS and PLA/CS-g-PLA samples presented in Figure 7c are in the same range as that required for a temporary cancellous bone substitute [25].

### 3.6. Biological Characterization

#### 3.6.1. Characterization of Osteoblast Cells Adhesion and Proliferation into PLA/CS Porous Composites Scaffolds

##### Osteoblast Cells Adhesion

For both PLA/CS and PLA/CS-g-PLA porous scaffolds, MG-63 osteoblast cells were seeded at 2 × 10^5^ and cultured for 24 h. Samples were then fixed in 4% paraformaldehyde overnight at 4 °C, then stained with Hoechst dye. Cells were visualized using an epifluorescence microscope. 

**Figure 8:**
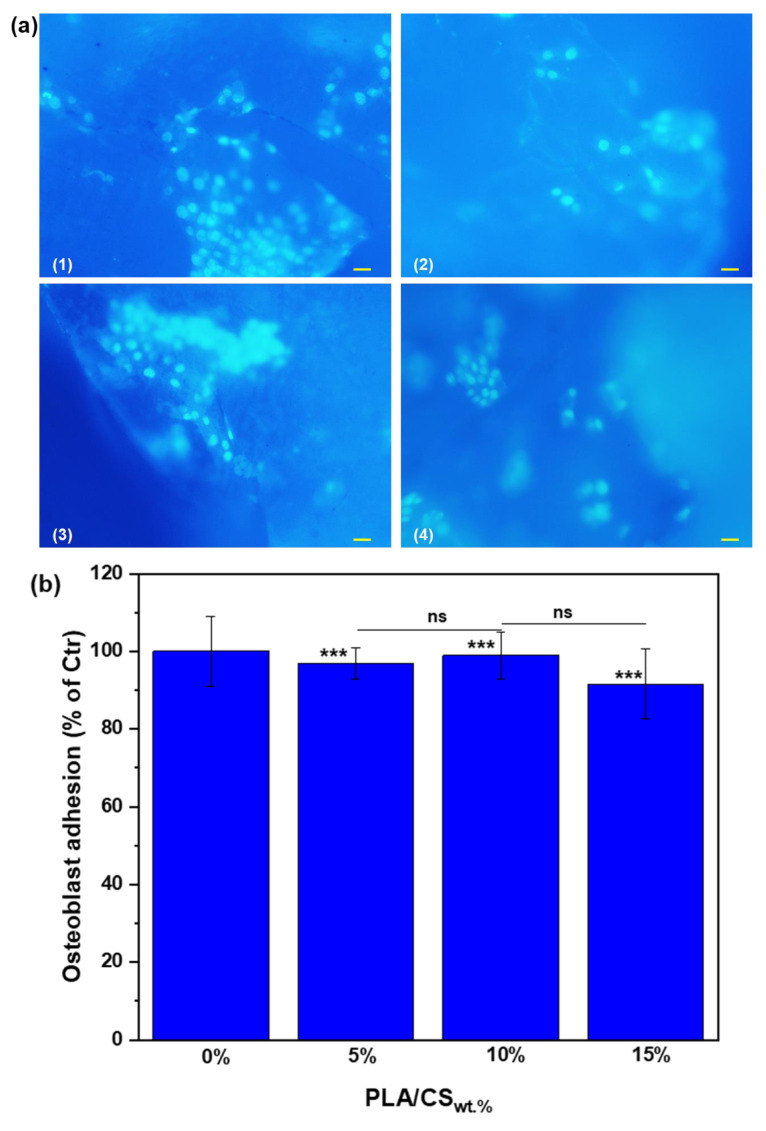
Osteoblast cells adhesion into pure PLA (Control, Ctr) and PLA/CS porous composite scaffolds: (**a**) Hoechst staining (qualitative); (**b**) MTS assays (quantitative). (Scale bar on the pictures = 50 μm), (*** *p* ˂ 0.001; ns = not significant).

The adhesion of the MG-63 osteoblast cells on the surface of the pores inside pure PLA and PLA/CS porous scaffolds was studied after 24 h incubation. Cell adhesion was evaluated qualitatively by means of Hoechst staining and quantitatively using an MTS colorimetric assay, as explained above in Section 2.3.6. The corresponding results are shown in Figure 8a,b. Figure 8a shows multiple illuminated dots corresponding to stained adherent cells. The osteoblasts cells have adhered well to the pure PLA scaffold (the control), as shown on picture (1), and the number of adherent cells is higher compared to PLA/CS composite scaffolds shown on pictures (2)–(4). These qualitative data were quantitatively confirmed by MTS colorimetric assay (Figure 8b), showing that the pure PLA porous scaffold (the control sample) presents slightly higher cell adhesion compared with the PLA/CS scaffolds. Such a decrease in cell adhesion with chitosan-loaded PLA could be due to the increased acidity of the culture medium. Indeed, as shown above in Figure 4a, the pH of the culture medium was below pH 7 for the PLA/CS composite scaffold. The decrease in pH is linked to the cationic properties in chitosan, as previously reported in the literature [43,44,46]. Furthermore, we noticed that chitosan-rich PLA scaffolds, especially the PLA/CS_15wt.%_, were too brittle and broke easily. Such poor mechanical properties, likely due to the long contact with culture medium, would decrease cell adhesion, as the scaffolds are degraded during the process of cell culture and become difficult to manipulate.

##### Osteoblast Cells Proliferation

**Figure 9:**
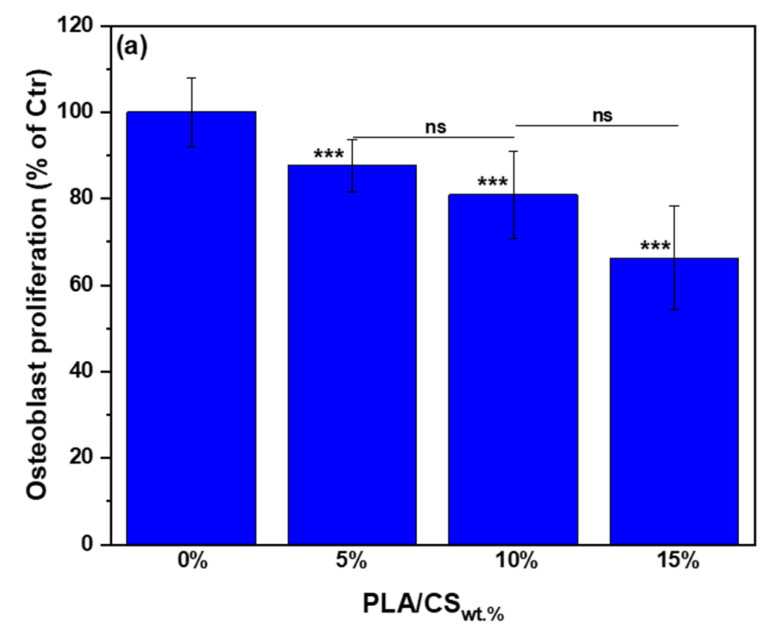

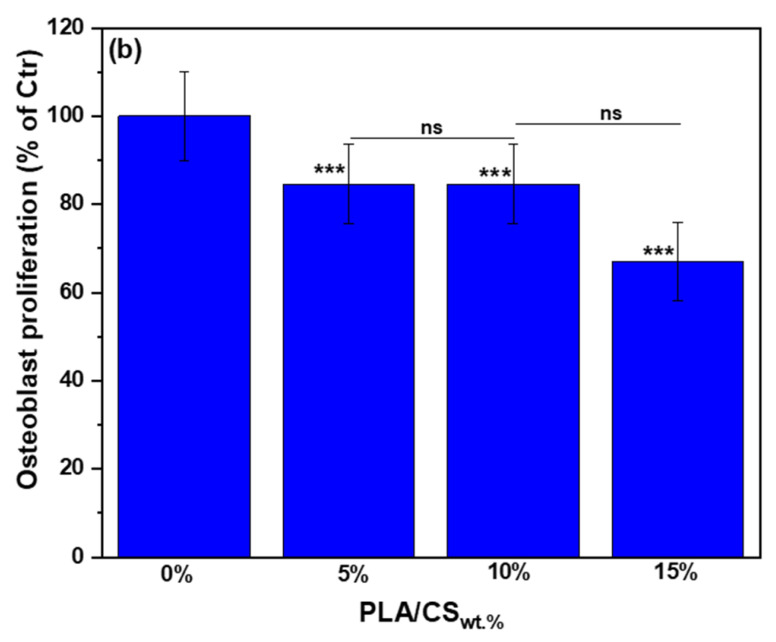
Osteoblast cells proliferation after 3 days (**a**) and 5 days (**b**) for pure PLA (Control, Ctr) and PLA/CS porous scaffolds at different CS contents. (*** *p* ˂ 0.001; ns = not significant).

To characterize the MG-63 osteoblast proliferation, the cells were seeded onto each scaffold at 1 × 10^5^ and cultured for 3 and 5 days. Cell proliferation was then evaluated by an MTS colorimetric assay, as explained in Section 2.3.6. The corresponding results after 3 and 5 days of cell culture are shown in Figure 9a,b, respectively. The results show that the absorbance for PLA/CS composite scaffolds is lower than that measured for pure PLA scaffolds (control sample). It decreases with increasing CS content from 5 to 15 wt.%, suggesting a decrease in the viable osteoblast cells number following the culture into the scaffolds for 3 and 5 days. Indeed, for the three CS contents studied (5, 10, and 15 wt.%), their corresponding PLA/CS composite scaffolds showed lower cell proliferation compared to pure PLA. As mentioned above, such a decrease could be due to the chitosan’s low pH and acidity [42,43,45]. In addition, we noticed that PLA/CS composite scaffolds, especially at 15 wt.% of CS, were relatively brittle, leading to degradation of the matrix even with careful handling. Such fragility may contribute to cell loss, thus, low absorbance after MTS assay.

#### 3.6.2. Characterization of Osteoblast Cells Adhesion and Proliferation into PLA/CS-g-PLA Porous Composites Scaffolds

##### Osteoblast Cells Adhesion

**Figure 10:**
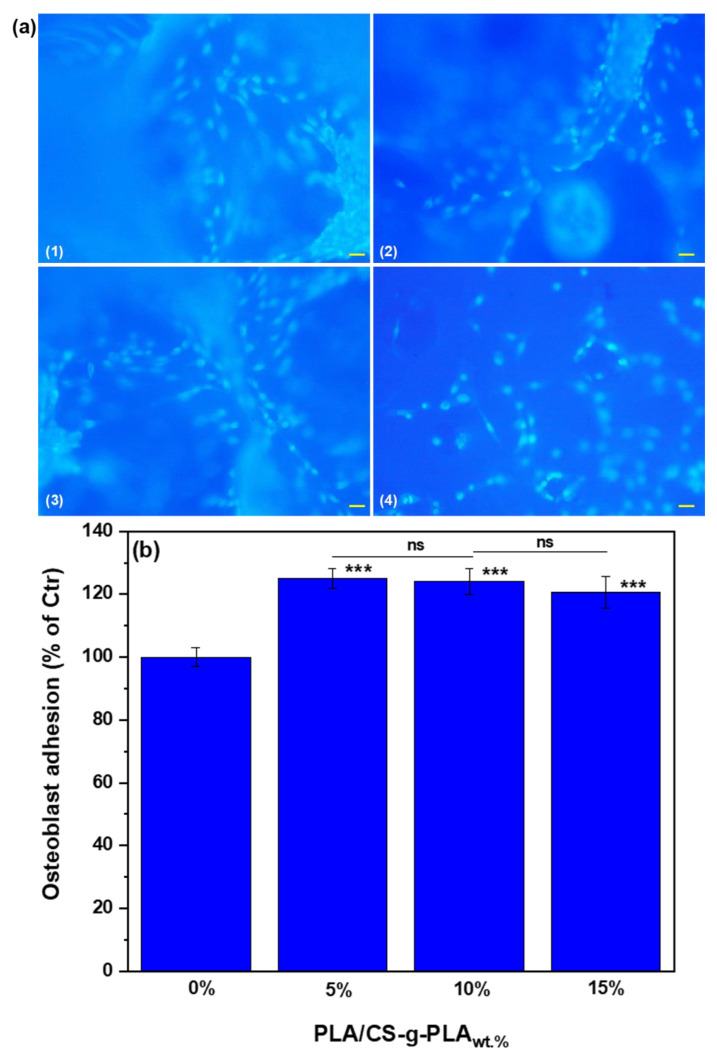
Osteoblast adhesion into PLA/CS-g-PLA porous scaffolds: (**a**) Hoechst staining; (**b**) MTS assays. (Scale bar on the pictures = 50 μm). (*** *p* ˂ 0.001; ns = not significant).

To elucidate the effect of chitosan compatibilization with PLA matrix on cells culture, the adhesion of the MG-63 osteoblast cells was studied for PLA/CS-g-PLA porous scaffolds, and the corresponding Hoechst staining and MTS assays results are shown in Figure 10 a,b, respectively. Contrarily to what was observed above for uncompatibilized PLA/CS composite scaffolds, picture (2) of Figure 10a, which corresponds to the compatibilized PLA/CS-g-PLA_5wt.%_ composite scaffold, shows a higher number of stained nuclei (that refer to adherent live cells), compared to the pure PLA scaffold (the control) shown in picture (1). Interestingly, for the two other CS-g-PLA higher contents (10 wt.% and 15 wt.%), more cell adhesion was also observed, as shown in pictures (3) and (4). These qualitative Hoechst staining results were quantitatively confirmed by the MTS assays, as shown in Figure 10b. Overall, chitosan compatibilization with PLA improved the adhesion of osteoblast cells. Such improvement in cell adhesion could be attributed to the hydrophilic nature of CS-g-PLA that promotes cells adhesion [49,50,51].

##### Osteoblast Cells Proliferation

**Figure 11:**
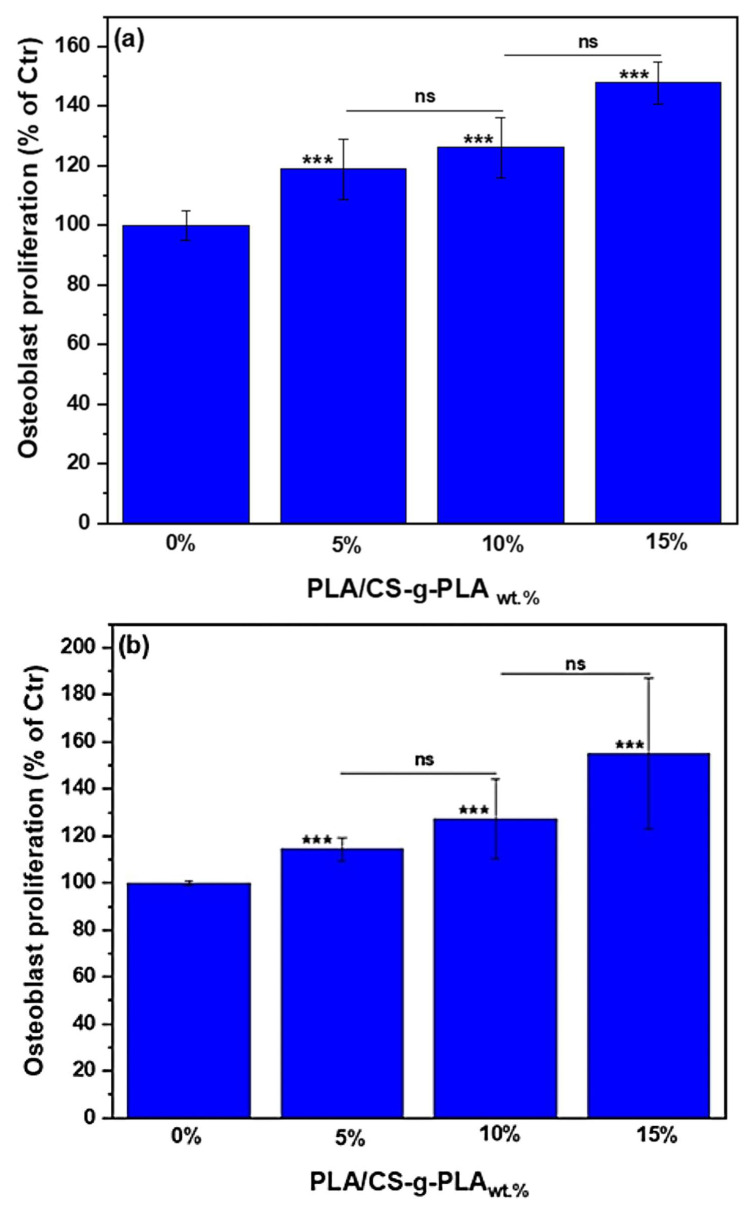
Osteoblast cells proliferation after 3 days (**a**) and 5 days (**b**) for pure PLA (Control, Ctr) and PLA/CS-g-PLA porous scaffolds at different CS-g-PLA contents. (*** *p* ˂ 0.001; ns = not significant).

Osteoblast cell proliferation was evaluated by an MTS colorimetric assay, as in the case of uncompatibilized PLA/CS composite scaffolds. The corresponding results after 3 and 5 days of cell culture are shown in Figure 11a,b, respectively. Contrarily to what was observed for uncompatibilized PLA/CS composite scaffolds following cell culture and MTS assays, compatibilized PLA/CS-g-PLA scaffolds showed increased absorbance levels with the presence of CS-g-PLA. Interestingly, cell proliferation was increased with the amount of grafted CS to PLA. Indeed, after 3 days, we noted that the higher the concentration of grafted chitosan, the greater was the cell proliferation (Figure 11b)). After 5 days, there was significant and much higher cell proliferation compared to PLA/CS scaffolds. These results indicate that the PLA/CS-g-PLA scaffolds are better for MG-63 osteoblasts cell proliferation [52,53].

## 4. Conclusions

In this study, we successfully developed by compression molding open-cell PLA/CS and PLA/CS-g-PLA composite scaffolds with three different CS and CS-g-PLA contents (5, 10, and 15 wt.%). We particularly focused on the compatibilization of CS with the PLA matrix by grafting PLA on CS backbone. Compatibilized PLA/CS-g-PLA scaffolds were then compared to the uncompatibilized PLA/CS scaffolds, particularly for their hydrolytic degradation and their abilities to adhere to osteoblast cells on the surface of their pores and to improve cell proliferation. It was observed that the immersion in PBS solution of PLA/CS composite scaffolds decreased the pH of PBS solution below pH 7.0 from the first day due to the cationic properties of CS. This was not the case for pure PLA and PLA/CS-g-PLA scaffolds, for which the change in the pH of PBS solution was very small and remained close to pH 7.0. This hydrolytic stability, mainly due to the improved interaction between the PLA matrix and CS-g-PLA copolymer, is very important to guarantee osteoblasts cell viability and makes PLA/CS-g-PLA scaffolds appropriate for bone regeneration. This was confirmed by the biological characterization of both uncompatibilized PLA/CS and compatibilized PLA/CS-g-PLA composite scaffolds. MTS colorimetric assay showed quantitatively that the adhesion and proliferation of MG-63 osteoblast cells were higher inside pure PLA scaffold compared with the uncompatibilized PLA/CS scaffolds due to the increased acidity of the culture medium. However, compatibilized PLA/CS-g-PLA scaffolds showed higher cell adhesion and proliferation than uncompatibilized PLA/CS scaffolds. Further, it was observed that cell proliferation increased with CS-g-PLA content and continued for up to five days of cell culture. 

## Figures and Tables

**Table 1:** Weight composition of PLA/CS and PLA/CS-g-PLA composites.

Sample Designation	PLA (wt.%)	ADA (wt.%)	CS or CS-g-PLA (wt.%)
PLA/CS_5wt.%_	88.1	6.9	5.0
PLA/CS_10wt.%_	83.1	6.9	10.0
PLA/CS_15wt.%_	78.1	6.9	15.0
PLA/CS-g-PLA_5wt.%_	88.1	6.9	5.0
PLA/CS-g-PLA_10wt.%_	83.1	6.9	10.0
PLA/CS-g-PLA_15wt.%_	78.1	6.9	15.0

## Data Availability

Not applicable.
